# COVID-19 pandemic–related emotional, social, and medical concerns of Latino patients with cancer: perspectives of mental health providers

**DOI:** 10.1097/or9.0000000000000124

**Published:** 2024

**Authors:** Rosario Costas-Muñiz, Maria F. Montana, Lourdes Ruda-Santolaria, Jose C. Sanchez-Ramirez, Normarie Torres-Blasco, Eida Castro-Figueroa, Loida Esenarro, Oscar Galindo-Vazquez, Cristiane Bergerot, Maria Claros, Bharat Narang, Jackie Finik, Francesca Gany, William Breitbart

**Affiliations:** a Department of Psychiatry & Behavioral Sciences, Memorial Sloan Kettering Cancer Center, New York, NY; b Department of Psychiatry, Weill Cornell Medical College, New York, NY; c Hospital Británico, Buenos Aires, Argentina; d Pontificia Universidad Católica del Perú, Lima, Perú; e Instituto Nacional de Salud, Lima, Perú; f Universidad de Lima, Lima, Perú; g Ponce Research Institute, Ponce Health Sciences University, Puerto Rico; h School of Medicine and School of Behavioral and Brain Sciences, Ponce Research Institute, Ponce Health Sciences University, Puerto Rico; i Universidad Peruana Unión, Lima, Perú; j Instituto Nacional de Cancerología (InCan), Mexico City, Mexico; k Centro de Cancer de Brasilia, Instituto Unity de Ensino e Pesquisa, Brasilia, DF, Brazil

## Abstract

**Introduction:**

Latino people with cancer might face additional health, emotional, and socioeconomic burdens of the COVID-19 pandemic.

**Methods:**

This study included data from two waves of (independent) assessments with providers of mental health services to Latino/Hispanic people with cancer from the United States, Spain, and Latin America (first wave: May-July 2020; second wave: March-July 2021) who completed a cross-sectional online survey with open- and closed-ended questions, including concerns of people with cancer with/without COVID-19.

**Results:**

The response rates were 15% for Wave 1 (N=88) and 14% for Wave 2 (N=115). For Wave 1, 74 surveys were completed by clinicians and included in the analyses; for Wave 2, 115 surveys were included. Providers (first [77%] and second [84%] waves) reported that most patients had concerns about stress/symptoms of anxiety, followed by concerns about COVID-19 exposure (first [74%] and second [82%] waves) and family members’ exposure (second wave 75%), hospital visits or appointments (82%, 79%, respectively), treatment/testing delays (69%, 72%, respectively), general health (58%, 71%, respectively), and income/salary loss or reduction (60%, 50%, respectively). According to providers, concerns of patients diagnosed with COVID-19 included fear of death and dying, spreading the disease, getting worse, and lack of appropriate medical care.

**Conclusion:**

Our findings reveal the need to address health, emotional, and socioeconomic burdens of the COVID-19 pandemic throughout Latin America, Spain and the United States for Latino people with cancer. Interventions targeting the healthcare access, emotional, and socioeconomic needs of Latino people with cancer are warranted.

## INTRODUCTION

The novel coronavirus 2019 (COVID-19) pandemic is one of the most critical global public health challenges for infectious disease management of the 21^st^ century so far. As of June 2023, there had been over 767 million cases and 6.9 million deaths globally and 193 million cases and 3 million deaths in the Americas.^1,2^ Due to public health and economic stability impacts, the pandemic presents a serious threat to mental health and healthcare access globally.^3–6 7,8^

A systematic review found that US Latino populations experienced disproportionately higher rates of severe COVID-19-related infection, hospitalization, and mortality than non-Latino White populations, but not higher case-fatality rates (mostly reported as in-hospital mortality),^9^ suggesting that healthcare access and exposure factors may underlie the observed disparities more than susceptibility due to comorbid conditions.^9^ Another study found that elevated incidence of severe COVID-19 among Latino individuals was due to higher infection rates, not increased susceptibility to progression.^10^ Thus, COVID-19 disparities most likely result from social, not biological, factors.^10^

Cancer patients report a significant impact of the pandemic on their care, essential needs, adjustment, and wellbeing. For people with cancer, the pandemic has affected cancer treatment continuity; it has caused follow-up appointment and diagnostic test delays and changes in the delivery of cancer care.^11–13^ Cancer-specific (active cancer) and other health-related factors (performance status, comorbidities) increase the risk of COVID-associated mortality for oncological patients, which may heighten infection fears.^14^ New York patients with ovarian cancer reported significant cancer worry (89%), anxiety (51%), and depression (27%) during the pandemic,^12^ with greater severity associated with younger age (<65 years), scheduled treatment or surgery, care delays, being immunocompromised (self-reported), and telemedicine use.^12^ In a systematic review^15^ of studies examining the psychological distress of cancer patients, the authors found that fear of COVID-19, fear of disease progression, disruption of oncology services, cancer stage, and immunocompromised status were the most common concerns or causes of psychological distress in oncology patients. In another review and meta-analysis^16^ of the depression and anxiety levels in cancer patients, the authors found high levels of depression and anxiety among people with cancer (prevalence of depression and anxiety exceeding more than 30% and cancer patients experiencing more anxiety than healthy controls) during the COVID-19 pandemic.

Latino individuals are exposed to several risk factors that impact their psychological adjustment and well-being. A US national survey showed that Latino people were more likely than non-Latino White people to experience factors associated with COVID-19 exposure (e.g., larger household sizes, in-person occupations, public transportation usage).^22^ Another found that Latino populations experienced high job loss and business closures.^23^ Latino respondents reported postponing education and health-related services, because of cost, and reported difficulty paying for housing.^23^ Latino adults were significantly more likely to experience housing instability related to the COVID-19 pandemic than non-Latino White and Black adults in the US city of Chicago in 2020 to 2021; pandemic-related housing instability was significantly associated with increased psychological distress among all races/ethnicities.^24^US Latino populations have experienced higher rates of food insecurity during the pandemic,^25^ and food insecurity is associated with poorer mental health and psychosocial stressors.^26^

Patients from low- and middle-income countries (LMICs) seemed to have been the most affected by the pandemic. In a survey conducted by the World Health Organization (WHO) with key informants from 105 countries, 90% of countries disrupted their essential health services when the COVID-19 pandemic began, and in 55% of them, cancer diagnosis and treatment were affected; the impact was greatest on LMICs.^27^ Further, in another study conducted by the WHO with informants from 130 countries, it was found that the pandemic also disrupted essential mental health care in 93% of the world’s countries.^28^ However, key informants revealed that in more than 80% of developed countries, mental health care continued via telehealth platforms, compared to less than 50% in developing countries.^29^

The literature has documented the impact of the pandemic on the care, essential needs, concerns, adjustment, and wellbeing of cancer patients. However, studies examining the impact of COVID-19 on the essential needs, adjustment, concerns, and well-being of Latino people with cancer are lacking. We explored the concerns and emotional, health-related, and socioeconomic needs of Latino people with cancer, including patients with COVID-19 diagnoses, as reported by mental health providers who treated Latino cancer patients throughout the US, Latin America, and Spain. Given the striking impact of the pandemic and disruption of care in LMICs, we conducted country-level sub-analyses. Mental health providers of cancer patients are the professionals whose main role is to explore and help manage the physical, psychological, social, and behavioral aspects of the cancer experience.^30^ Further, mental health professionals who treat Latino cancer patients have reported addressing concerns and coping strategies pertinent to issues of family, emotions, experience with cancer and its trajectory, end of life, communication, education, socioeconomic factors, and physical symptoms.^31^ Given the scope of mental health providers’ work and professional experiences, they can be key informants about the concerns and emotional, health-related, and socioeconomic needs of Latino cancer patients. The main strength of this article is to provide longitudinal data on adult Latino people with cancer about the impact of the COVID-19 pandemic in 2020 and 2021 from the perspective of mental health professionals.

## METHODS

### Participants

Mental health providers who participated in our survey were members of the FIPOL (*Formación de Investigación Psicosocial Oncológica Latinoamericana;* Capacity on Psychosocial Oncology Research for Latin America) network (www.fipol.info). The FIPOL listserve includes clinicians and researchers interested in psychosocial oncology who work with Latino people with cancer in the US, Puerto Rico, Spain, and Latin America. The providers were identified through psycho-oncology newsgroups, professional organization membership rosters, LinkedIn, and cancer center mental health facility websites and were invited to join FIPOL.^32^

All FIPOL listserve members were invited by email to participate in the web-based *Practice of Psychosocial Oncology During the COVID-19 Pandemic* cross-sectional survey. In the first wave (May-June 2020), we invited 585 providers to participate; 224 accessed the survey, of whom 53 did not complete it, and 83 were ineligible; 88 providers completed the survey, yielding a response rate of 15%. Seventy-four were clinicians and were included in analyses for Wave 1. In the second survey wave (March-July 2021), we invited 824 providers to participate; 357 accessed the survey, of whom 110 did not complete it, and 132 were ineligible; 115 providers completed the survey, yielding a response rate of 14%.

### Procedures

Survey invitations included a study description, inclusion criteria, and a survey link. Responses were anonymous. Providers completed an eligibility screener and were eligible if they were professionals working in the mental health field (e.g., psychiatrists, psychologists, social workers, counselors, and other mental health professionals) and actively provided mental health services to Latino/Hispanic people with cancer in Spanish and/or Portuguese. Eligible individuals were (1) mental health professionals, (2) actively providing mental health services, (3) to Latino/Hispanic people with cancer, (4) in Spanish and/or Portuguese. Participants provided online consent, received a downloadable information letter, and, if agreeable, completed the survey. The study was approved as exempt research by Memorial Sloan Kettering Cancer Center’s institutional review board (protocol number X20–028).

### Measures

The provider survey elicited demographic (age, gender), practice (years of experience), and training (specialized training/courses) characteristics. The survey was divided in two sections. The first section included 18 items, and 4 of them were available only for Wave 2; the second section included 14 questions. The questions were similar, but in the first section, providers reported the magnitude of patients affected by the concerns (i.e., none, few, some, most, or all patients), and in the second section, providers reported the magnitude of patient distress for each of 14 concerns on a Likert-like scale (1=least distressful, 5=most distressful). The items explored the proportion and distress levels of patients with pandemic-related concerns: emotional (2 questions: stress, depression), interpersonal (2 questions: feeling lonely or alone, increased family tension, kids’ reactions), health-related (3 questions: personal/family contagion fear), healthcare access (6 questions: attending the hospital, cancer treatment or testing delays, general health concerns, access to healthcare, technology access for healthcare, accessing healthcare resources in patient’s language) and socioeconomic (4 questions: income loss, food insecurity, paying utilities, housing insecurity) concerns. One additional item was open ended (other additional concerns?); the qualitative responses are described in the Results section. In Wave 2, the four added items were: exposure from family members, loneliness while staying at home, increased family tension or conflict, and kids’ reactions. The 15- to 20-minute survey was available in Spanish and English in Waves 1 and 2 and also Portuguese in Wave 2. The original survey was developed in Spanish, based on a literature review and structured feedback from the FIPOL steering committee members. Per our IRB standard process, the survey was then translated into Portuguese and English by bilingual translators. Two independent translators (for each language) performed quality check reviews.

### Analyses

Descriptive statistics examined participants’ responses to demographic, academic, clinical practice background questions, and responses to the magnitude of patients expressing concerns and, subsequently, their distress levels. Because of missing data, the number of respondents to specific questions was noted. Analyses were conducted with the individual items; they were not aggregated for indexes or scales. Chi squares were conducted to compare magnitude of patients expressing concerns and distress level of providers practicing in high-income countries and middle-income countries following the World Bank classification system,^33^ which classifies countries according to their region, lending status, and economy. For 2023, the World Bank classified 28 countries as low-income, 108 as middle-income, and 81 as high-income countries.^33^The responses to the open-ended questions were coded, by three coders, with thematic content analysis.^32^ Using Excel, the coders first open-coded the responses and then met to generate final codes and the codebook^34–36^; they re-coded the responses using the final codes, held consensus meetings to discuss the coded responses, reached agreement, and quantified the coded responses for reporting purposes. The frequency of concerns reported in the open-ended questions and the closed-ended COVID-19 patient concerns were quantified and summarized.

## RESULTS

Of survey Wave 1 providers (n=74), most identified as Latino/Hispanic (96%), and all offered services in Spanish (Table 1). Most identified as White (52%). Most practiced in Mexico (27%), Peru (14%), US (11%), Argentina (11%), and Puerto Rico (7%). Providers often had master’s (36%) or doctoral (29%) degrees; 33% had practiced for ≥15 years, and 48% treated 11 to 30 Latino people with cancer per week. Of Wave 2 providers (n=115), most provided services in Spanish (93%) and identified as White (59%). Most practiced in Mexico (25%), Argentina (18%), Spain (11%), or Peru (8%). Most had a master’s (33%) or doctoral (22%) degree; 39% had been in clinical practice for ≥15 years, and 59% treated one to ten Latino people with cancer per week.

In Wave 1, three-quarters (76%) of providers reported actively treating patients either in person (51%) and/or remotely (75%), by phone (62%) and/or through videoconferencing (63%). However, in Wave 2, most (90%) reported actively treating patients less frequently in person (21%) and more frequently remotely (81%), by phone (56%) and/or through videoconferencing (77%).

Some of the pandemic-related patient concerns reported by providers in Waves 1 and 2 were similar and included attending clinic/hospital appointments (Wave 1, 82%; Wave 2, 79%), exposure concerns (74%, 82%, respectively), treatment/testing delays (69%, 72%, respectively) (Table 2). However, providers reported more patients in Wave 1 than Wave 2 being concerned with food insecurity (32% vs. 20%) and income loss (60% vs. 50%) and reported more patients in Wave 2 than Wave 1 experiencing pandemic-related stress/anxiety (84% vs. 77%) and general health concerns (71% vs. 58%).

In Waves 1 and 2, providers reported patients’ most distressing concerns as COVID-19 exposure (Wave 1, mean 4.3 [0.9 SD]; Wave 2, mean 4.4 [0.9 SD]), family member exposure (mean 4.1 [1.0 SD], mean 4.3 [0.9 SD], respectively), attending their healthcare appointments (mean 4.1 [1.0 SD], mean 4.3 [1.0 SD], respectively), treatment/testing delays (mean 4.1 [1.0 SD], mean 4.2 [1.1 SD], respectively), general health (mean 3.9 [1.1 SD], mean 4.0 [1.1 SD], respectively), income/salary loss (mean 3.6 [1.3 SD], mean 3.3 [1.3 SD], respectively), and being alone/lonely (mean 3.5 [1.2 SD], mean 3.3 [1.3 SD], respectively) (Table 3).

In Waves 1 and 2, there were no statistically significant differences in distress levels for the listed concerns between high-income (29% of sample: USA, Spain, Puerto Rico, Chile, and Uruguay) and middle-income (71% of sample) countries (HICs and MICs; as classified by the World Bank) and between providers from the US and providers from all other countries. In Wave 1, providers from Latin America/Spain reported that more patients (40%) than reported by US providers (25%) were concerned about family member exposure to COVID-19 (*X*^2^=7.65, *p*=.02). In Wave 2, all US providers (100%) reported that most patients were concerned about feeling lonely or being alone while staying at home compared to 44% of providers from Latin America/Spain (*X*^2^=6.4, *p*=.04). Also, they reported that more patients were concerned about accessing resources and services in their language (100%) than providers from Latin America/Spain (21%, *X*^2^=16.9, *p*=.001). Providers from HICs reported that more patients were concerned about housing insecurity (rent or mortgage, 86%) than providers from MICs (60%, *X*^2^=8.5, p=.02).

In Wave 1, 37% of providers had treated patients diagnosed with COVID-19 in person (8%) or remotely (30%). Qualitatively reported patient concerns (Table 4) included death and dying (n=7), spreading the disease (n=7), and spreading the disease to family (n=5). In Wave 2, 57% of providers had treated patients diagnosed with COVID-19, in person (49%) or remotely (38%). Qualitatively reported patient concerns included spreading COVID-19 to family/others (n=14), COVID reinfection (n-9), death and dying (n=7), and getting worse/immunosuppression (n=7).

## DISCUSSION

In this study of the concerns of Latino people with cancer during the COVID-19 pandemic, mental health providers in the US, Latin America, and Spain, reported that their patients experienced high levels of stress and anxiety and were most concerned about COVID-19 exposure (of patients and family members) and medical care delays. Other concerns reported by approximately half of each sample were concerns about patients’ reduced income, access to healthcare, and feelings of loneliness and depression. These findings are particularly relevant given the high prevalence of COVID-19 in US Latino populations.^9^

Providers across the US, Latin America, and Spain reported similar levels of pandemic-related stress and anxiety among their patients. The World Health Organization has found that the primary mental health concern/need during COVID-19 is elevated stress and anxiety precipitated by the health threat and mitigation measures (quarantines, social distancing).^28^ Our study highlights broad commonalities in the international experience of Latino people with cancer. There were some differences in potential sources of anxiety. Providers reported significantly fewer US Latino patients to be worried about family member COVID-19 exposure than patients in Latin American/Spain. Providers also reported that significantly more US Latino patients experienced loneliness or isolation during the pandemic than those in Latin America/Spain.

A group of very distressing concerns among people with cancer (with/without COVID-19) was treatment continuation and healthcare access during the pandemic. Social distancing and stay-at-home orders limited access to general and mental healthcare, particularly in-person care. Mental healthcare and access to telehealth and the internet have been revealed as pandemic critical needs; however, Latinos and patients with lower socioeconomic status have less service access in the US and Latin America.^37,38^ Although telehealth and other remote solutions for this care have been widely offered and used, many patients face technological and financial barriers that might exacerbate health outcome disparities influenced by technological disparities.^39–41^ More efforts are needed to improve general and mental health service delivery, delivery of evidence-based practice, and internet/telehealth availability.^37,42,43^ Additionally, accessing healthcare in their language was a frequent healthcare access concern among US Latino patients, according to the provider surveys. Oncology providers need to consider the impact of social inequality and concerns when caring for their patients.

Income concerns among people with cancer have been associated with worry, stress, and reduced quality of life.^44–46^ Financial distress could increase disease and death risk and lead Latino patients to skip or delay mental and medical healthcare because of costs.^47^ Results of our provider surveys suggested that housing concerns were significantly more frequent among patients in HICs than MICs. From 2020 to 2021, providers reported decreased patient food insecurity and loss of income concerns and increased pandemic-related stress/anxiety and general health concerns. It is possible that more patients were concerned with basic needs (food and housing) and income at the beginning of the pandemic than later when healthcare access became a greater concern. In the US, hardships related to meeting basic expenses, paying rent, and food insufficiency generally lessened from August 2020 through August 2021, although they were still present for many.^48^

Providers perceived the fears of spreading the disease, death and dying, and getting worse, and access to care as critical concerns for patients diagnosed with COVID-19. Several studies have shown possible long-term mental health effects of COVID-19 infection, including clinical mood symptoms.^49–51^ Mental healthcare is an imperative and critical need during the pandemic. In people with cancer, these cognitive and mental health complications can be more complex and critical due to the frequent feelings of vulnerability and health threat cancer patients face.

Clinicians and healthcare teams can address patients’ financial, emotional, and practical issues by screening and exploring patients’ economic pressures and how they might impact their cancer treatment adherence or overall adjustment, refer patients to patient navigators and case managers to assist them with their financial needs, and/or to community-based agencies for financial and practical support.^52^ Interventions aiming to improve health equity and decrease cancer disparities, especially during the pandemic, should include comprehensive programs that address patients’ emotional, practical, logistical, and socioeconomic needs. These programs should incorporate a screening phase that includes a comprehensive evaluation of such needs, followed by a management plan.

### Limitations

A significant limitation was the low response rate of 15%, and although 15% is an acceptable response rate for a rapid-response brief online survey, there are generalizability and interpretation concerns. Our findings might not be generalizable to all mental health providers treating cancer patients in Latin America, Spain, and the US given the low response rate. Comparison analyses were also limited by the small sample of providers practicing in the US. Additionally, the validity of the findings could also be affected by providing information “by proxy”; in other words, providers were reporting on the perceived distress levels of their patients, which may have been affected by “recall bias” and lack of accuracy of retrieved information. Furthermore, the information retrieved might have been influenced by cases that consumed more energy or time in the clinic versus reflecting the actual magnitude of those affected or of distress-level perceptions. Research into the promotion and delivery of mental healthcare for people with cancer through telehealth and information on the practice, setting, and patients that the providers typically serve is needed.

## Conclusion

The social and emotional burden, fears, and anxiety related to the spread of COVID-19, and the impact of measures taken (social distancing, stay-at-home recommendations) have revealed the need for wide availability of mental healthcare through remote options in Latin America and the US for Latino people with cancer.

## Figures and Tables

**Table 1.** Characteristics of mental health provider survey participants from the US, Spain, and Latin America^a^^,^^b^

Characteristic	Providers, First Wave^c^ (N=74), No. (%)	Providers, Second Wave^d^ (N=115), No. (%)
**Age, y (mean, SD)**		
**Gender**		
Female	56 (81.2)	95 (83.3)
Male	13 (18.8)	19 (16.7)
**Hispanic or Latino**	66 (95.7)	115 (100.0)
**Race**		
White or Caucasian	36 (52.2)	68 (59.1)
Black	1 (1.4)	2 (1.7)
Mixed	8 (11.6)	45 (39.1)
Asian	1 (1.4)	
**Preferred language**		
English	11 (12.2)	5 (4.3)
Spanish	79 (87.8)	107 (93.0)
Portuguese		3 (2.6)
**Practice Place**		
Mexico	19 (27.1)	29 (25.2)
Dominican Republic	0 (0.0)	1 (0.9)
United States	8 (11.4)	4 (3.5)
Puerto Rico	5 (7.1)	6 (5.2)
Ecuador	2 (2.9)	4 (3.5)
Colombia	3 (4.3)	5 (4.3)
Panama	3 (4.3)	2 (1.7)
Honduras		
Peru	10 (14.3)	10 (8.7)
Argentina	8 (11.4)	21 (18.3)
Venezuela	1 (1.4)	2 (1.7)
Guatemala		2 (1.7)
El Salvador		1 (0.9)
Chile	4 (5.7)	5 (4.3)
Spain	3 (4.3)	13 (11.3)
Costa Rica	2 (2.9)	3 (2.6)
Brazil	1 (1.4)	3 (2.6)
Paraguay	1 (1.4)	2 (1.7)
Uruguay	1 (1.4)	2 (1.7)
Bolivia	1 (1.4)	
**Academic degree**		
Licensed psychologist with bachelor’s degree	8 (11.6)	31 (27.0)
Master’s in psychology	22 (31.9)	36 (31.3)
Master’s in social work	2 (2.9)	0 (0.0)
Master’s in counseling	1 (1.4)	3 (2.6)
PhD or PsyD in psychology	20 (29.0)	23 (21.8)
MD in psychiatry	7 (10.1)	2 (1.7)
Postgraduate degrees and fellowships	7 (10.1)	18 (15.7)
**Years of licensure, mean (SD)**	15.1 (9.9)	16.5 (10.5)
**Time in clinical practice, y**		
1-10	28 (40.6)	45 (40.5)
11–15	18 (26.1)	23 (20.7)
≥15	23 (33.3)	43 (38.7)
**No. of weekly cancer patients**		
1–10	23 (33.3)	62 (59.0)
11–30	33 (47.8)	29 (27.6)
≥31	13 (18.8)	14 (13.3)

a Percentages may not equal 100% due to rounding

b Missing values were excluded from the analyses

c First wave: May-July 2020

d Second wave: March-July 2021

**Table 2.** Quantity of Latino cancer patients reporting COVID-19–related concerns from the mental health providers’ perspectives^a^,^b^

Patient Concerns	Providers, First Wave^c^ (N=74), No. (%)	Providers, Second Wave^d^ (N=115), No. (%)
**Stress or symptoms of anxiety related to the COVID-19 situation**		
None	2 (2.7)	
A few to some	15 (20.3)	18 (15.9)
Most or all of them	57 (77.0)	95 (84.1)

**Concern about COVID-19 exposure**		
None	1 (1.4)	1 (0.9)
A few to some	18 (24.3)	19 (17.0)
Most or all of them	55 (74.3)	92 (82.1)

**Concern about coming to the hospital during the COVID-19 situation**		
None	1 (1.4)	2 (1.8)
A few to some	11 (14.9)	21 (18.9)
Most or all of them	61 (82.4)	88 (79.3)

**Concern about family members’ exposure to COVID-19**		
None		2 (1.8)
A few to some		26 (23.4)
Most or all of them		83 (74.8)

**Concerns about treatment or testing being delayed due to the COVID-19 situation**		
None	2 (2.7)	4 (3.6)
A few to some	21 (28.4)	27 (24.5)
Most or all of them	51 (68.9)	79 (71.8)

**General health concerns (medication, primary care visits, etc.)**		
None	4 (5.4)	2 (1.8)
A few to some	26 (35.1)	30 (27.0)
Most or all of them	43 (58.1)	79 (71.2)

**Worried about income or salary (loss or reduction)**		
None	1 (1.4)	4 (3.7)
A few to some	28 (37.8)	50 (46.7)
Most or all of them	44 (59.5)	53 (49.5)

**Access to healthcare**		
None	6 (8.1)	
A few to some	28 (37.8)	
Most or all of them	39 (52.7)	

**Concern about feeling lonely or being alone while staying at home**		
None		9 (8.1)
A few to some		51 (45.9)
Most or all of them		51 (45.9)

**Symptoms of depression related to the COVID-19 situation**		
None	2 (2.7)	7 (6.3)
A few to some	45 (60.8)	59 (53.2)
Most or all of them	27 (36.5)	45 (40.5)

**Concern about increased tension or family conflict while staying at home**		
None		10 (9.0)
A few to some		70 (63.1)
Most or all of them		31 (27.9)

**Concern about kids’ reactions, behavior, and home education while staying at home**		
None		8 (7.8)
A few to some		70 (68.0)
Most or all of them		25 (24.3)

**Food access or insecurity (lack of availability of appropriate food)**		
None	15 (20.3)	38 (35.2)
A few to some	34 (45.9)	48 (44.4)
Most or all of them	24 (32.4)	22 (20.4)

**Concern about paying utilities (e.g., electricity, water, phone) or other bills**		
None	7 (9.5)	27 (25.0)
A few to some	50 (67.6)	61 (56.5)
Most or all of them	16 (21.6)	20 (18.5)

**Housing insecurity (rent or mortgage)**		
None	12 (16.2)	36 (33.3)
A few to some	47 (63.5)	56 (51.9)
Most or all of them	14 (18.9)	16 (14.8)

**Technology access concerns (telehealth and access to virtual health services)**		
None	10 (13.5)	24 (22.6)
A few to some	49 (66.2)	66 (62.3)
Most or all of them	9 (12.2)	16 (15.1)

**Concern about accessing resources and services in their language**		
None	23 (31.1)	66 (68.8)
A few to some	25 (33.8)	19 (19.8)
Most or all of them	7 (9.5)	11 (11.5)

a Percentages may not equal 100% due to rounding

b Missing values were excluded from the analyses

c First wave: May-July 2020

d Second wave: March-July 2021

**Table 3.** Distress levels of Latino cancer patients reporting COVID-19-related concerns from mental health providers’ perspectives^a^^,^^b^^,^^c^

Patient Concerns	Distress Level Score	
	Providers, First Wave^d^ (n=68), Mean (SD)	Providers, Second Wave^e^ (N=115), Mean (SD)
**Concern about exposure to COVID-19**	4.3 (0.9)	4.4 (0.9)
**Concern about family members exposure to COVID-19**	4.1 (1.0)	4.3 (0.9)
**Concern about coming to the hospital during the COVID-19 situation**	4.1 (1.0)	4.3 (1.0)
**Concerns about treatment or testing being delayed due to the COVID-19 situation**	4.1 (1.0)	4.2 (1.1)
**General health concerns (medication, medical visits, etc.)**	3.9 (1.1)	4.0 (1.1)
**Worried about loss or reduction of income or salary **	3.6 (1.3)	3.3 (1.3)
**Feeling lonely or being alone while staying at home**	3.5 (1.2)	3.3 (1.3)
**Concern about increased family tension while staying at home**	3.2 (1.2)	3.1 (1.1)
**Concern about kids’ reactions or homeschooling**	3.2 (1.2)	2.7 (1.3)
**Concern about paying utilities (e.g., electricity, water, phone) or other bills**	3.0 (1.3)	2.8 (1.4)
**Food insecurity (lack of availability of appropriate food)**	3.0 (1.4)	2.6 (1.3)
**Housing insecurity (rent or mortgage)**	2.8 (1.4)	2.7 (1.3)
**Technology access concerns (telehealth and access to virtual health services)**	2.6 (1.3)	2.5 (1.2)
**Concern about accessing resources and services in their language**	1.9 (1.4)	2.0 (1.3)


a Percentages may not equal 100% due to rounding

b Missing values were excluded from the analyses

c Scored on a Likert-like scale ranging from 1, least distressful, to 5, most distressful

d First wave: May-July 2020

e Second wave: March-July 2021

**Table 4.** Patient (diagnosed with COVID-19) concerns as reported qualitatively by providers (in responses to open-ended questions)

Patient Concerns	No.
**Survey Wave 1 (May-July 2020) (N=74)**	
Death and dying	7
Spreading the disease	7
Spreading the disease to family	5
Lack of appropriate medical attention or substandard at-home care	3
Ability to manage or control emotions/fear of emotional breakdown	3
Isolation-related stress	3
Family member worry	2
Job/income loss	2
COVID-19 impact on cancer/treatment	2
COVID-19 stigma	2
Uncertainty	1
Catastrophic thoughts	1
Symptom control	1
Viral load	1
Getting worse/immunosuppression	1
**Survey Wave 2 (March-July 2021) (N=115)**	
Spreading the disease to family/others	14
COVID reinfection	9
Death and dying	7
Getting worse/immunosuppression	7
Isolation-related stress	5
COVID-19 impact on cancer/treatment	5
Not being able to receive in-person care/telehealth concerns	4
Ability to manage or control emotions/fear of emotional breakdown	4
Lack of appropriate medical attention or substandard at-home care	1
Advanced directives	1
